# Authors' Reply

**DOI:** 10.1371/journal.pmed.0030284

**Published:** 2006-06-27

**Authors:** Peter J Hotez, David H Molyneux, Alan Fenwick, Eric Ottesen, Sonia Ehrlich Sachs, Jeffrey D Sachs

**Affiliations:** **1**The George Washington UniversityDepartment of Microbiology and Tropical Medicine, Washington, District of ColumbiaUnited States of America; **2**Liverpool School of Tropical MedicineLymphatic Filariasis Support Centre, LiverpoolUnited Kingdom; **3**Imperial College LondonInfectious Disease Epidemiology, St. Mary's Campus, LondonUnited Kingdom; **4**Emory University, Rollins School of Public HealthAtlanta, GeorgiaUnited States of America; **5**Columbia University, The Earth InstituteNew York, New YorkUnited States of America

We appreciate the comments by our colleagues from the Drugs for Neglected Diseases initiative (DNDi) highlighting the importance of research and development programs for a new generation of control tools (e.g., drugs, diagnostics, vaccines, surveillance instruments) to combat the neglected tropical diseases [ 1]. Indeed, we have great admiration for the outstanding track record of the DNDi along with its sister organizations, including the Institute for One World Health, TDR-WHO, and the Seattle Biomedical Research Institute, as well as a small but distinguished community of academic and government scientists.

In both our paper cited by Torreele et al. [ 2] and an earlier companion paper also published in *PLoS Medicine* [ 3], we went to some lengths to point out that the ultimate elimination of some of the most burdensome endemic neglected tropical diseases will likely require more than simply innovations in preventative chemotherapy, such as our proposed rapid-impact, pro-poor package. Such achievements, especially for diseases such as Buruli ulcer, hookworm, human African trypanosomiasis, and leishmaniasis, will almost certainly also require advances in biotechnology leading to the development and distribution of new drugs and vaccines. It is also for that reason that each of the biomedical scientists who co-authored the *PLoS Medicine* papers has devoted his or her lifetime to research on neglected tropical diseases and has contributed to the development of new control tools for hookworm, lymphatic filariasis, malaria, onchocerciasis, schistosomiasis, and other diseases of poverty. In addition, Jeffrey Sachs, the health economist on our project, previously led an international call to establish a US$1.5 billion Global Health Research Fund, to finance basic and applied research on the diseases of poverty [ 4]. In short, we completely agree with the plea by Torreele et al. to embrace research and development as an essential component for any global neglected tropical disease initiative.

The major points of our *PLoS Medicine* articles are these: (1) the disease burden of neglected tropical diseases has been underestimated and this group of diseases may be as important as HIV/AIDS, malaria, and tuberculosis; (2) there is a moral imperative to recognize the plight of the world's most impoverished who suffer from neglected tropical diseases; and (3) beginning today, we can make a rapid impact on the lives of these populations through an effective, sustainable, rapid, and highly cost-effective intervention package of donated drugs.

Ultimately, control and elimination of some of our most devastating neglected tropical diseases will likely require additional biotechnological solutions. Even then, this will require careful integration of the new with the old, along the lines of the vaccine-linked chemotherapy strategies recently proposed by Bergquist et al. [ 5]. Therefore, we need to do our very best to achieve sustainable morbidity reductions by using the donated drugs we have in hand today, with the understanding that success in this endeavor must not lead to complacency. We know all too well how the emergence of chloroquine and DDT resistance derailed global malaria eradication efforts during the 1960s [ 6] and that we must be ready to simultaneously champion research as well as implementation. We also believe that it would be unethical in the context of the timeframe of the Millennium Development Goals to ignore what we can do for poor people now at such a low cost [ 7], as we know that research takes time to come to fruition in terms of products, policies, financing, and practice. Drugs of proven efficacy and quality are available now for some of the neglected tropical diseases, and they can be delivered despite the resource constraints in African health systems. It is gratifying to see a number of countries are now prioritizing the control or elimination of neglected tropical diseases as a national policy and are establishing budget lines to ensure sustainable implementation of the tools we have. There have been too many public health failures in the past—and so it is essential that we take the real opportunity to act on behalf of the world's poor.
